# Acute sensory and motor response to 45-s heavy isometric holds for the plantar flexors in patients with Achilles tendinopathy

**DOI:** 10.1007/s00167-018-5050-z

**Published:** 2018-07-04

**Authors:** S. O’Neill, J. Radia, K. Bird, M. S. Rathleff, T. Bandholm, M. Jorgensen, K. Thorborg

**Affiliations:** 1 School of Allied Health, University of Leicester, Leicester, UK; 2 Research Unit for General Practice in Aalborg, Department of Clinical Medicine, Aalborg University, Aalborg, Denmark; 3 Department of Occupational Therapy and Physiotherapy, Aalborg University Hospital, Aalborg University, Aalborg, Denmark; 4 Physical Medicine and Rehabilitation Research-Copenhagen (PMR-C), Department of Physical and Occupational Therapy, Clinical Research Center, Amager-Hvidovre Hospital, University of Copenhagen, Copenhagen, Denmark; 5 Department of Orthopedic Surgery, Clinical Research Center, Amager-Hvidovre Hospital, University of Copenhagen, Copenhagen, Denmark; 6 Department of Geriatrics, Aalborg University Hospital, Aalborg, Denmark; 7 Sport Orthopedic Research Center-Copenhagen (SORC-C), Amager-Hvidovre University Hospital, Copenhagen, Denmark

**Keywords:** Achilles, Tendinopathy, Isometrics, Rehabilitation, Loading, Pain

## Abstract

**Purpose:**

The purpose of this study was to explore the immediate effects of heavy isometric plantar flexor exercise on sensory output (pain during a functional task and mechanical pain sensitivity) and motor output (plantar flexor torque) in individuals with Achilles tendinopathy.

**Methods:**

Sixteen subjects with Achilles tendinopathy participated in the study, mean (SD) age 48.6 (8.9) years and Victorian institute assessment-Achilles (VISA-A) score 61.3 (23.0). Sensory testing assessing pain during a functional task, mechanical pain sensitivity and motor output, and plantar flexor peak torque was completed prior to the intervention. All subjects completed a 45-s heavy isometric plantar flexor contraction and were then re-tested using the same sensory and motor tests. Motor output was assessed using isokinetic dynamometry at speeds previously identified as of interest in subjects with Achilles tendinopathy.

**Results:**

Only 9 of the 16 subjects experienced pain during a functional task, self-reported pain was 4.2 (1.9) numerical rating scale (NRS) pre-intervention and 4.9 (3.2) NRS postintervention (n.s.). Mechanical pressure sensitivity was 446.5 (± 248.5) g/mm^2^ pre-intervention and 411.8 (± 211.8) g/mm^2^ post-intervention (n.s.). Mean concentric plantar flexor torque at 90 and 225°/s was 47.1 (14.5) and 33.6 (11.6) Nm, respectively, pre-intervention and 53.0 (18.5) and 33.4 (6.6) Nm post-intervention (*p* = 0.039 and n.s.). Eccentric torque at 90°/s was 98.5 (34.2) Nm preintervention versus 106.0 (41.4) Nm post-intervention (n.s.).

**Conclusion:**

In this exploratory study, patients with Achilles tendinopathy had a varied sensory and motor output response to heavy isometric contractions. Using the recommended approach of heavy 45-s isometric contractions did not offer a meaningful acute benefit for sensory or motor output for subjects with Achilles tendinopathy. Based on this study, heavy 45-s isometric contractions cannot be recommended for immediate pain relief or improved motor output for patients with Achilles tendinopathy.

**Level of evidence:**

IV, prospective cohort study.

## Introduction

Successful treatment of Achilles and patella tendinopathy has for the last three decades focussed on eccentric resistance training [1, 3, 15, 44]. Recent evidence has challenged the need for pure eccentric contractions and current recommendation is that heavy slow resistance exercise is of equal value and may even improve patient satisfaction [6, 26]. Evidence from systematic reviews supports these treatment options for both Achilles and patellar tendinopathy [31, 43]. From 2015 to 2016, preliminary evidence from studies on patellar tendinopathy suggests an immediate improvement in pain after heavy isometric contractions and an improvement in motor output [40, 41]. Rio et al. used heavy isometric holds at 70% of maximal voluntary contraction (MVC) in athletes with patellar tendinopathy and reported an immediate reduction in pain during a single-leg decline squat. Subject’s pain reduced from 7/10 to 0/10 on a numerical rating scale (NRS) and this pain reduction lasted for at least 45 min. Pain reductions of this magnitude and longevity have obvious benefits in clinical practice where individuals may then be able to participate in activities previously limited due to pain. The reported increases in motor output may also expedite a return to normal activities, the mechanism by which this may occur is thought to be a reduction in cortical inhibition [40].

In the most recent edition of Brukner and Khan’s “Clinical sports medicine: Injuries” there is a section highlighting isometric exercises as “stage 1” rehabilitation for patients with Achilles tendon pain [10]. In this evidence-based sports medicine textbook, it is suggested that progression to stage 2 (isotonic exercises) should not be started until individuals are competent at isometrics and can sustain 45-s contractions steadily with an external load. In addition, it is also suggested that isometric plantar flexor exercises may increase motor output via a reduction of cortical motor inhibition [10, 39, 40, 48]. Research has yet to test whether isometric contractions have the ability to reduce pain and increase motor output immediately in patients with Achilles tendinopathy. It is important that new interventions are tested to ensure efficacy and effectiveness prior to clinical implementation.

The purpose of this study was to explore the immediate effects of heavy isometric plantar flexor exercise on sensory output (pain during a functional task and mechanical pain sensitivity) and motor output (plantar flexor torque) in individuals with Achilles tendinopathy.

The hypothesis was that heavy isometric contractions of the plantar flexors will result in significant reductions in subject’s pain whilst increasing motor output.

## Materials and methods

This is a prospective cohort study where outcome assessments were performed at baseline and repeated immediately after the intervention. No comparator was used, and the study was considered exploratory, being the first to investigate the preliminary efficacy of this approach on self-reported pain, mechanical pressure pain thresholds and motor output when applied to the Achilles tendon. The reporting of the present study follows the STROBE and [49] TIDieR guidelines [20] supplemented with mechano-biological exercise descriptors suggested by Toigo & Boutellier [47].

Recruitment used a snowball sampling method and involved contacting local running clubs to identify individuals suffering from “Achilles tendon” pain [7]. Interested individuals were invited into the university to undergo a clinical examination to determine if they were eligible.

### Inclusion criteria

Inclusion criteria included a diagnosis of unilateral mid-portion Achilles tendinopathy based on previously identified criteria, see Table 1, with symptoms for greater than 3 months, minimum age of 18 with no upper age limit and an ability to give informed consent.

**Table 1:** Criteria used to diagnose Achilles tendinopathy, all criteria needed to be present for a diagnosis of Achilles tendinopathy to be made

Localied Achilles tendon pain
Pain provoked by physical activities in a dose-dependent way, i.e. running activities provoke pain more than walking
Pain remains after completion of exercise and will reduce over time but is aggravated with the next loading session/activity
Reproduction of pain with palpation of the tendon [21, 37]
Positive London hospital test and/or painful arc sign of the Achilles tendon [28, 37]
Identification of ultrasonographic features, such as increased diameter and hypoechoic regions in keeping with a diagnosis of Achilles tendinopathy [28, 34, 38]

### Exclusion criteria

The exclusion criteria was set to exclude Plantaris-induced Achilles tendinopathy, Paratendonitis, Fascia Crura tears, Longitudinal tears/splits or partial ruptures as these sub-categories of Achilles tendinopathy are thought to be provoked by loading-based rehabilitation [32, 51]. The assessment of these criteria was undertaken during a clinical examination and ultrasound tissue characterization (UTC) (UTC imaging, The Netherlands) scan, at present there is debate around the diagnosis of these sub-categories but the consensus is that tears present with a sudden onset of pain [24, 33, 51]. Therefore, subjects with sudden onset of pain were excluded. Plantaris-induced tendinopathy was diagnosed based on medial-sided-only Achilles tendon pain and UTC imaging confirming medial-located tendon changes in the absence of changes in other areas of the tendon. The UTC scan was completed and analysed by the same experienced user (S O’N) using previously identified protocols [16, 33]. Subjects with mild severity indicated by a Victorian institute of sports assessment: Achilles (VISA-A) score above 90 points were excluded [22].

Subjects were also excluded if they had received or were currently undergoing treatment for their tendinopathy or had other lower limb musculoskeletal, vascular or neurological disorders. The exclusion of subjects who had or were already receiving therapy was to ensure selection bias was not introduced by including subjects experiencing failure or benefit of previous loading-based exercise therapy and to limit expectation bias in subjects.

### Measures of sensory and motor output

#### Sensory output

##### Self-reported pain during a functional task

Pain during an aggravating activity was considered the primary outcome based on the work by Rio et al. [40]. Assessment of load-based tendon pain was based on routine clinical tests, progressing through greater loads. Reliability of this particular measurement strategy was not performed but these tests form part of the VISA-A which has been shown to be reliable [22, 42]. All subjects initially completed 10 bilateral heel raises, if these were pain free they completed 10 unilateral heel raises, if these were pain free then 10 unilateral hops were completed, if subjects experienced pain at any time the test was stopped and subjects were asked to score their pain using an 11-point NRS with 0 being no pain and 10 being maximal imaginable pain [18]. All tests were completed on a flat surface and all subjects were required to move into end-range plantar flexion during the heel raises. If all tests were pain free, the subject was deemed to score 0. If subjects completed all three tests without pain, further attempts to induce functional activity-related pain were not pursued, for ethical reasons. Pain score and the task and number of repetitions subjects completed were recorded, the same task was used post-intervention to determine whether their pain during a functional task had improved.

##### Mechanical pressure pain thresholds

Mechanical pressure pain threshold was quantified using an electronic Von Frey’s device (Electronic Von Frey Anesthesiometer, IITC, USA), this tool has been shown to have good reliability, Lin coefficient above 0.81 and is accurate to 1 g [46] The site of each subject’s pain was marked during the clinical examination based on the site of most tenderness on palpation. This area was where all mechanical pressure sensitivity readings were taken. The average of three readings was used for each measurement.

#### Motor output

**Fig. 1:**
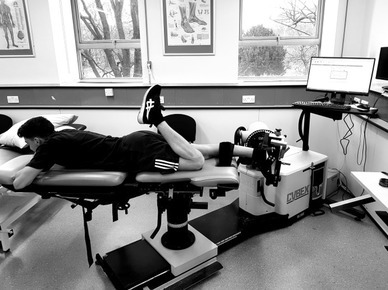
The position used for isokinetic dynamometry testing. The test leg was fully extended. The non-tested leg (left) was only flexed in this image to allow visualisation of the leg being tested

Isokinetic dynamometry (Humac Norm, CSMI solutions, USA) utilised previously identified speeds and test positions [3, 4, 35]. The testing used an extended knee position (Fig. 1) rather than a flexed knee position as this position has greater test–re-test reliability, with the intra-class correlation coefficient (ICC) between 0.70 and 0.87 [5]. Three test speeds were used in two different contraction modes. Concentric torque was measured in 90°/s and 225°/s and eccentric torque was measured at 90°/s [5].

#### The intervention

**Fig. 2:**
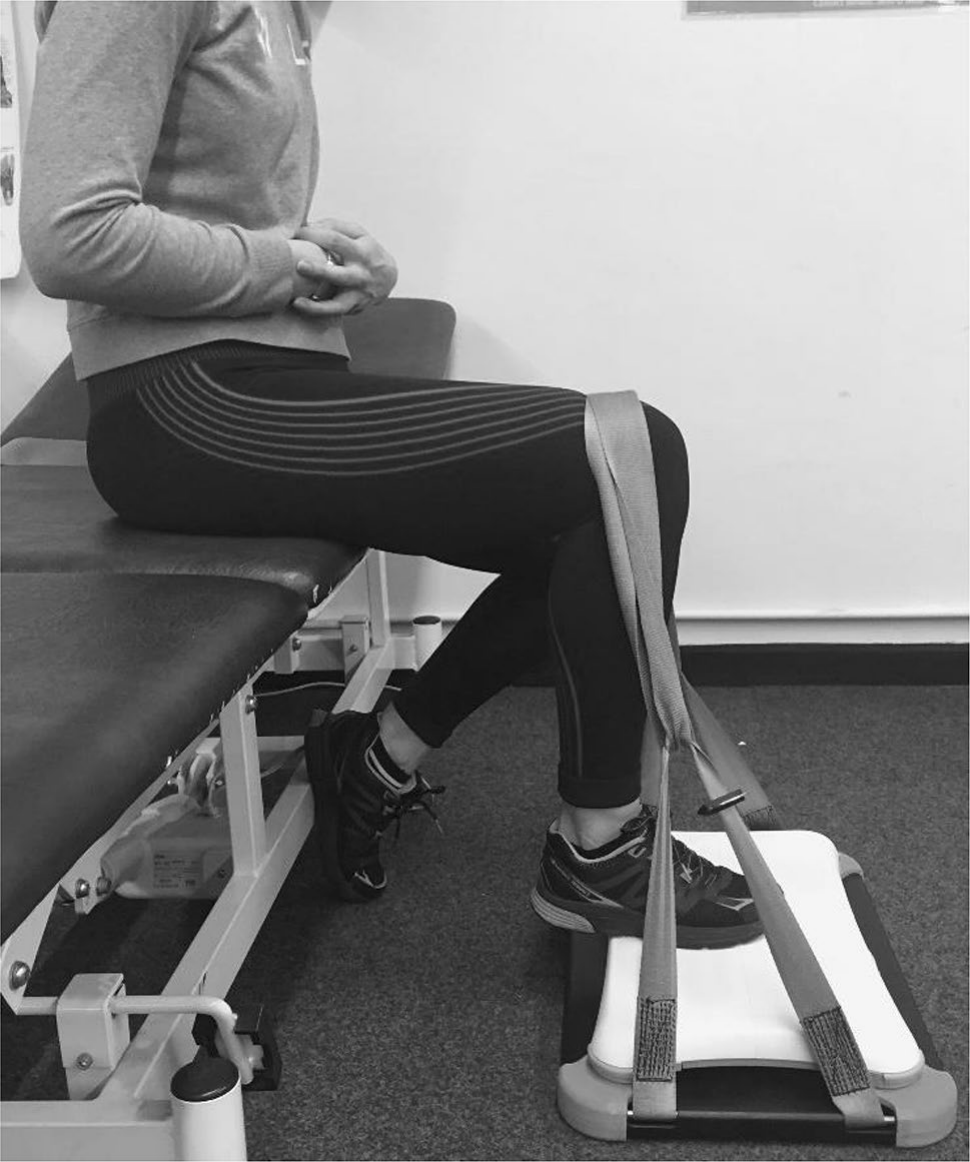
The Fysiometer setup with the subject seated on a physiotherapy couch, the hip and knee at 90° flexion and the ankle joint in 10° of plantar flexion. The Wii platform was positioned flat on the floor and a belt with a tensioner device was used to fix the ankle in position for the isometric contraction

The intervention was based on previous work by Rio et al. [40, 41] and recommendations for Achilles tendinopathy [10] The intervention utilised five 45-s isometric plantar flexor contractions at 70% MVC—separated by 2-min rests in accordance with the protocol used for patellar tendinopathy [10, 40, 41]. To determine subject’s 70% MVC all subjects had their MVC (isometric) measured using a Fysiometer (Fysiometer ApS, Denmark) [19]. The Fysiometer software utilises a sampling rate of 100hz which is filtered through a fourth-order Butterworth filter, the software has an accuracy of 0.1 kg on the whole measurement range from 0 to 300 kg. The test–re-test ICC is 0.79, based on a study of 20 subjects completed at the university. Isometric force data were only used as a method to determine the intervention threshold. Mean (SD) isometric force output during the pre-intervention was 101.9 kg (± 23.6), this equated to 127.7 (± 29.4) percent of body weight (%BW). Isometric data were not measured post-intervention as it has not previously been shown to be relevant. The Fysiometer has previously been identified as a valid and reliable tool for quantifying maximal isometric plantar flexor muscle strength [19]. Each subject was carefully positioned with their heel over the nearside edge of the Wii platform and their metatarsals on the inside edge of the unit (Fig. 2). All subjects were seated on a plinth with the ankle positioned at 10° of plantar flexion and the knee and hip at 90° flexion, a goniometer was used to verify these positions. The flexed knee position was utilised for the isometric intervention in this study as our previous work has identified that the Soleus muscle may be more involved in the motor deficits associated with Achilles tendinopathy and the area of the Achilles most commonly affected by tendinopathy appears to relate to fascicles linked to Soleus [13, 17, 45]. The subject’s own running trainers were worn for all testing to reduce any discomfort caused by the Wii platform. Subjects underwent a ramped series of plantar flexor contractions to establish their MVC, this consisted of three gradual contractions for familiarisation prior to three MVCs, with the peak force being recorded. Standardised verbal encouragement was offered to all subjects during the MVC test. The peak MVC from this test was used to determine 70% MVC for the intervention. Real-time visual feedback of contraction force was provided by the Fysiometer software, with force along the vertical axis and time along the horizontal axis, the subject’s contraction levels were represented as a bold trace between the two axes. The computer cursor was moved along the horizontal axis to help subjects visualise their 70% MVC target. Verbal encouragement was given when necessary, this allowed the subjects to modify their force output in real time ensuring they maintained a contradiction at 70% MVC for the duration of the test intervention.

The intervention was completed on the Fysiometer using the test position as described for the strength test. The 10 degrees of plantar flexion was chosen as a manageable position for all subjects as piloting of the test had revealed that some subjects cannot maintain a coordinated contraction in full plantar flexion without excessive muscle fasciculation. Excessive muscle fasciculation is not considered beneficial for tendinopathy and may re-enforce faulty motor patterning or be responsible for greater tendon loading [10, 36, 39]. The information given to all the subjects was standardised in an attempt to reduce any expectation bias, each subject had the same verbal statement given to them regarding the study “We are going to test a new exercise for Achilles tendon pain, an Australian group have shown this to be excellent for knee tendons (patellar tendon pain) and we want to see if it helps Achilles tendon pain”. This statement was given by the same experienced physiotherapist and attempts were made to ensure the intonation, gramma and inflection were standardised across all subjects in an attempt to reduce the effect of non-specific treatment effects. No attempt was made to blind the subjects to the study hypothesis as it was made clear to them that the study was assessing whether isometric exercises affected their tendon pain and/or altered their motor output.

Ethical approval was granted by the University of Leicester (UK) ethics board and consent was obtained from all participants before enrollment in the study, approval ID 3989-so59-medical education.

### Statistical analysis

A sample size estimation was completed based upon the NRS data from Rio et al. [40], which reported a reduction of 7 points after heavy isometric contractions; we, therefore, anticipated a similar response in this study. The power was set as 80% with a two-tailed significance of 5%; the calculation revealed a sample size of four subjects would be required. A sample size calculation was also completed for isokinetic strength measures using previously published minimal detectable change (MDC) values, this estimate used the same power and significance levels [5]. The number of subjects varied between 6 and 10 depending on which element of isokinetic strength was assessed—concentric or eccentric, respectively. To account for any subject failing parts of the testing, we aimed to include approximately 15 subjects.

Statistical analysis was completed to compare means at baseline to post-intervention. Data were found to be normally distributed using visual inspection of histograms and Shapiro–Wilks test. A paired *t* test was used as the data were normally distributed. The analyses aimed to explore if the intervention reduced pain sensitivity (pain during functional activity and mechanical pressure pain threshold) and altered motor output. Analyses were made using the available data (no imputations were made for missing data points); this was displayed graphically using the raw data.

## Results

A total of 20 individuals responded to the study invitation, 19 attended for examination and one was unable to arrange a suitable appointment. All subjects were examined; two subjects were excluded as they did not have a diagnosis of Achilles tendinopathy. A further subject was excluded as their Achilles tendinopathy was considered to be related to Plantaris involvement. The decisions to exclude subjects were made prior to collecting data. In total, 16 individuals (5 female) met the inclusion criteria. Demographic data regarding subjects can be viewed in Table 2.

**Table 2:** Demographic data (*n* = 16, 5 females)

Age, years	48.6 (± 8.9)
Height, cm	173.4 (9.1)
Weight, kg	81.6 (± 14.3)
VISA-A score, points	61.3 (± 23.0)
Duration of symptoms, months	46.5 (± 55.2; 3.5 to 177.8)

Values are means (± 1SD) for all variables except duration of symptoms, which is reported as (± 1SD; range)

### Sensory output

#### Self-reported pain during a functional task

**Fig. 3:**
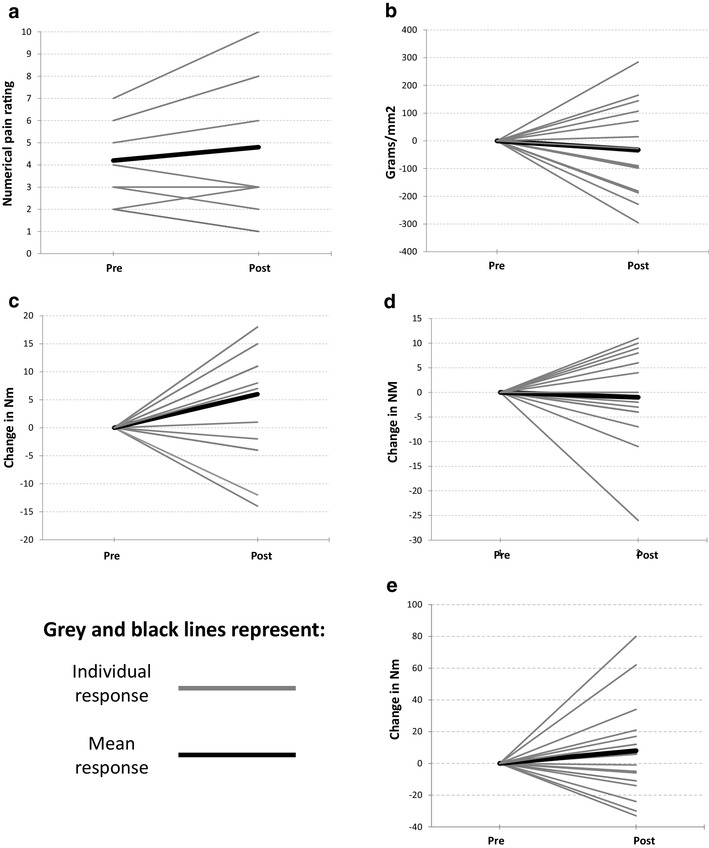
**a**–**e** Acute effects of isometrics in Achilles tendinopathy;* PF* plantar flexor and* Nm* Newton meters

Only 9 of the 16 participants reported pain during either heel raises (bilateral or unilateral) or hopping on the day of examination. The mean change in self-reported pain during a functional task is shown in Table 3 and the individual responses for all subjects are shown in Fig. 3a, these changes were not significant. Since some subjects did not experience pain during functional testing it was deemed appropriate to assess for differences in baseline variables to determine if there was an explanation for this difference. No between-group differences where identified for any variable when comparing those with self-reported pain on functional testing and those without except VISA-A score where it was observed those with pain during functional testing had a lower (worse) score [54.3 (± 15.8)] compared with those without pain on functional testing [70.3 (± 9.3)].

**Table 3:** Changes in each measurement variable from pre- to post-intervention

Measurement variable, unit	Pre-intervention mean (± 1SD)	Post-intervention mean (± 1SD)	Change mean (95% CI)
Sensory output			
Pain during a functional task, NRS-points	4.2 (± 1.9)	4.9 (± 3.2)	− 0.7 (− 1.82–0.49)
Pressure pain threshold, g/mm^2^	446.5 (± 248.5)	411.8 (± 211.8)	34.8 (− 49.2–118.8)
Motor output			
Maximal CON torque at 90°/s, Nm	47.1 (± 14.5)	53.0 (± 18.5)	− 5.9 (− 11.4–0.33)
Maximal CON torque at 225°/s, Nm	33.6 (± 11.6)	33.4 (± 6.6)	0.2 (− 4.8–5.3)
Maximal ECC torque at 90°/s, Nm	98.5 (± 34.2)	106.0 (± 41.4)	− 7.5 (− 24.1–9.1)

*CON* concentric; *ECC* eccentric; *NRS* numerical rating scale

*Statistically significant change from pre- to post-intervention based on a paired *t* test

#### Mechanical pressure pain thresholds

Mean mechanical pressure pain thresholds reduced non-significantly as a result of the intervention as shown in Table 3, a decrease of − 32.4 g/mm2 (95%CI − 46.3–111.0). Figure 3b shows the individual response in mechanical pressure sensitivity to the intervention.

### Motor output

#### Isokinetic dynamometry

Mean maximal concentric plantar flexor torque at 90°/s increased significantly (Table 3) as a result of the intervention. No significant change was observed in either the concentric 225°/s or the eccentric 90°/s tests. Figure 3c–e shows the individual response of subject’s plantar flexor torque to the intervention.

## Discussion

The most important finding of the present study is the lack of pain relief with heavy 45-s isometric plantar flexor contractions and the identification of a variable response with some subjects having minor benefit and others significantly worsening. It is important to acknowledge this is the first study examining the immediate effects of heavy 45-s isometric plantar flexor contractions on sensory and motor output in individuals with Achilles tendinopathy. The results demonstrate that subjects who experienced pain during the functional tests did not experience a statistically significant change in pain as a result of the intervention at a group level. The graphical representation (Fig. 3A) highlights the individual responses to the intervention with those with higher NRS scores experiencing an increase in pain. Individuals with the highest NRS scores appeared to be worsened whilst those below the mean had some benefit. Subject’s with lower NRS scores (< 4) experienced reductions in pain, but only of 1 point. A reduction of 2 points has previously been identified as being the minimally important clinical difference, questioning whether the observed change can be considered clinically important [18]. The functional tests were not painful for all subjects, with only nine individuals being symptomatic with the tests.

The mean mechanical pressure pain thresholds did not alter significantly, but individual variations were observable with changes occurring in both directions. The reliability of pressure pain threshold has been shown to be “good” in patella tendinopathy [52] but has not been examined for Achilles tendinopathy. To our knowledge, there is no previous identification of the minimum clinically important difference (MCID) for mechanical pressure sensitivity for the Achilles tendon. MCID is the minimal amount by which a measurement needs to change to be considered clinically meaningful for a patient. The lack of predetermined MCID makes it difficult to consider whether some individuals may have experienced a meaningful change in mechanical pressure sensitivity. Mean motor output did not change for plantar flexor concentric 225°/s or eccentric 90°/s torque; however, there was a statistical change in concentric 90°/s torque. Despite mean concentric plantar flexor torque at 90°/s increasing by a statistically significant value, this value did not exceed the MDC of the measure (12.7 Nm) and is, therefore, unlikely to represent an actual measurable change [5]. Visual inspection of Fig. 3c shows that only two subjects improved above the MDC, suggesting these individuals had meaningful changes to motor output. Rio et al. previously reported increased motor output post-isometric loading, our results contrast with theirs as the changes in motor output were not statistically significant except for the concentric 90°/s measurement and this did not exceed the MDC and, therefore, does not represent an actual change in force. This lack of a response may be because cortical excitation is present in Achilles tendinopathy [8] as opposed to the cortical inhibition observed in patella tendinopathy [40]. The findings of this study suggest that isometric exercises do not acutely alter either self-reported pain during a functional task, mechanical pressure pain thresholds or motor output to a statistically or clinically relevant extent.

The different results observed for patellar and Achilles tendinopathy [5, 40] may be due to the different tendons under examination or the uncertainty and lack of statistical robustness from preliminary studies including very few subjects. However, our findings are similar to those of Coombes et al. [12] who examined the effects of two different levels of isometric exercise for lateral epicondylalgia. Their findings suggested that the response to isometric exercise is highly individual as can be observed in our study. This highlights that caution should be applied in suggesting widespread clinical implementation and usage of isometric treatment of tendinopathy in general, and in Achilles tendinopathy particularly, when we already have treatments with level 1 evidence of long-term effectiveness [25, 27, 31, 43, 50].

Only a subgroup of our subjects (9/16) experienced pain during their functional examination, this is not uncommon as subjects with Achilles tendinopathy have often reduced their aggravating activities prior to examination. It is also possible that our testing program, with the maximum tendon load being unilateral hops, was not sufficient to trigger pain in these subjects. However, it was considered unethical to induce tendon pain using greater loads, i.e. 5-km run.

### Methodological considerations and limitations

Despite the chosen test position negatively affecting gastrocnemius force production [5, 9, 14, 23], subjects were able to generate mean isometric forces of 127.7% BW (± 29.4). This level of force production is important as current proponents of heavy isometric interventions for tendinopathy suggest heavy loads are crucial for analgesia [10, 39]. Higher loads would not have been achieved using extended knee positions for the intervention as our previous work on runners with and without Achilles tendinopathy found peak torque occurred in flexed knee positions rather than the expected knee extended positions. That is evidenced in this study when a comparison of the concentric and eccentric torque values is made with the isometric data, this shows greater forces occurred during the isometric test (flexed knee) versus the isokinetic test (extended knee). It would be possible to use different ankle positions but this would limit force production further and, therefore, tendon load and motor cortex drive as the muscle unit would be moved away from mid-range, a position where most muscles generate peak force. Increased dorsiflexion would cause some degree of compression of the Achilles insertion and possibly produce pain limiting subject’s ability to maintain a 70%MVC in this position. Due to these elements, test positions of 5–10° plantar flexion are probably most useful. Our study clearly highlights that despite heavy isometric loads through the Achilles tendon, subject’s experienced limited analgesic responses.

The findings of this study are limited to mid-portion Achilles tendinopathy and the intervention protocol tested. Different muscle activation thresholds (%MVC), and test positions (knee and ankle) may produce different effects; however, it would be unclear why this may be the case. The current suggestion for isometrics is they should be very heavy and in positions where the tendon has little compression, both aspects being adhered to in the protocol used. However, it is also possible that different tendons respond in different ways, particularly the Achilles and patella, where tendinopathy causes different tissue changes: the patella becomes stiffer whilst the Achilles becomes less stiff, this may affect the response to isometric loading [11]. It may be the different sub-categories of Achilles tendinopathy excluded from this study would also respond differently to the intervention. However, the group of participants included in this study represents the typical patient with Achilles tendinopathy seen in sports medicine/physiotherapy departments [2, 3, 6, 29, 30].

Other than the differences in the tendon under examination there are large differences in the ages of the populations within the studies. The study on lateral epicondylalgia by Coombes et al. [12] included subjects with a mean age of 52.0 years while the studies by Rio et al. [40, 41] on patella tendinopathy included subjects with a mean age of 26.9 and 22.5 years. The current study included subjects with a mean age of 48.6 years. Whilst the ages differ across the studies they reflect the typical age groups afflicted by the different tendinopathies. It is conceivable that age or other variables we are as yet unaware of may explain the observed differences in outcome. However, since the present study utilised typically afflicted patients (age, presentation and disability) it is possible to conclude that isometric loads seem to offer little promise for immediate analgesia in the typical Achilles tendinopathy patient.

## Conclusion

In this exploratory study, patients with Achilles tendinopathy had a varied sensory and motor output response to heavy 45-s isometric contractions. Using the recommended approach of heavy isometric contractions did not offer a meaningful acute benefit for sensory or motor output in the majority of the included subjects with Achilles tendinopathy. Based on data from the present study, heavy 45-s isometric contractions cannot be recommended for immediate pain relief or improved motor output as first-stage rehabilitation for patients with Achilles tendinopathy.
